# Evaluating the repellent effect of four botanicals against two *Bactrocera* species on mangoes

**DOI:** 10.7717/peerj.8537

**Published:** 2020-03-04

**Authors:** Waqar Jaleel, Desen Wang, Yanyuan Lei, Guojun Qi, Ting Chen, Syed Arif Hussain Rizvi, Veeran Sethuraman, Yurong He, Lihua Lu

**Affiliations:** 1 Key Laboratory of Bio-Pesticide Innovation and Application, Guangdong Province, Guangzhou, China; 2 Engineering Research Center of Biological Control, Ministry of Education, Guangzhou, China; 3 Department of Entomology, College of Agriculture, South China Agricultural University, Guangzhou, Guangdong Province, China; 4 Plant Protection Research Institute, Guangdong Academy of Agricultural Sciences, Guangzhou, Guangdong Province, China; 5 Guangzhou Key Laboratory of Insect Development Regulation and Application Research, Institute of Insect Science and Technology & School of Life Sciences, South China Normal University, Guangzhou, China; 6 Key Laboratory of Natural Pesticide and Chemical Biology, Ministry of Education, South China Agricultural University, Guangzhou, China

**Keywords:** Bactrocera, *Seriphidium brevifolium*, *Azadirachta indica*, *Piper nigrum*, Repellent, Quercetin

## Abstract

**Background:**

*Bactrocera dorsalis* and *B. correcta* are economically important fruit fly pests of crops, vegetables, fruits, and nuts worldwide, especially in China. Nowadays in China, *B. correcta* is a second notorious pest of many fruits after *B. dorsalis*. Different botanicals have been tested against the *B. dorsalis* but in the case of *B. correcta*, no records were published.

**Methodology:**

This study evaluated the repellency of four botanicals (*Seriphidium brevifolium*, *Piper nigrum*, *Azadirachta indica* and quercetin) in acetone dilutions (5%, 2.5% and 1%) against the *B. dorsalis* and *B. correcta* at the laboratory conditions (25 ± 2 °C, 60 ± 5% relative humidity, and a photoperiod of L:D 14:10 h).

**Results:**

The number of visits after 24–48 h, oviposition punctures, and pupae made by both species were lower on the treated mangoes in comparison to untreated mangoes. *S. brevifolium*, *P. nigrum*, *A. indica* and quercetin have significantly reduced the visits, ovipositional punctures, and pupae of both species. Among botanicals, the *P. nigrum* was the most effective repellent against *B. correcta* and as well as *B. dorsalis*. However, the harmful effects of these botanicals against natural enemies are still unknown.

## Introduction

Mangoes are important fruits all over the world especially in Asia because of delicious taste and essential nutrient content (Sial et al., 2015). According to FAO reports (2017), mangoes were produced approximately 50 million tons worldwide, while in China was approximately 4.8 million tons (FAO, IFAD, UNICEF, WFP & WHO, 2017).

*Bactrocera dorsalis* (Hendel) and *B. correcta* (Bezzi) are serious threats to fruits especially mangoes all over the world (Ekesi et al., 2016; Jaleel, He & Lü, 2019; Jaleel et al., 2018a). Nowadays in China, the *B. correcta* is a second notorious pest of different fruits after *B. dorsalis*. Female *Bactrocera* flies directly lay eggs inside the mango skin by ovipositor (Allwood et al., 1999; Ekesi et al., 2016; Jaleel, Lu & He, 2018; Jaleel et al., 2018a).

Several studies have been reported the management of *Bactrocera* species using pesticides (Daane & Johnson, 2010; Jin et al., 2011; Nadeem et al., 2014). Moreover, pesticides residues in fruits are the major concern regarding human health (Amaro & Godinho, 2012). A number of population of *B. dorsalis* have evolved high levels of resistance towards nearly all commonly used insecticide groups (Jin et al., 2011). Many studies have been reported high resistance in field strains of *B. dorsalis* to trichlorfon (Jin et al., 2011; Khan & Akram, 2018). However, farmers need more reliable and safer control methods to prevents the attack of *Bactrocera* species worldwide, especially in China (Khan et al., 2017).

Alternatively, botanicals are more reliable, readily biodegradable, and less risk of resistance development in *Bactrocera* flies (Campos et al., 2018; De Oliveira et al., 2014; Isman, 2006; Khan et al., 2017). Botanicals are economically cheap in production (Siskos, Konstantopoulou & Mazomenos, 2009; Weaver et al., 1997). Botanicals are mostly specific in nature and have less impact on the survival of natural enemies (Potts et al., 2016). Therefore, botanicals pesticides are more reliable control methods against *Bactrocera* species in the Integrated Pest Management program (Ilyas, Khan & Qadir, 2017; Hikal, Baeshen & Said-Al Ahl, 2017; Khan et al., 2017; Naumann & Isman, 1995).

Several studies have been reported the repellency of several botanical extracts against different *Bactrocera* flies for example, *B. zonata* (Saunders), *B. oleae* (Rossi) (Ilyas, Khan & Qadir, 2017; Rehman et al., 2009a, 2009b; Siddiqi et al., 2006), *B. cucurbitae*, and *B. dorsalis* (Chen et al., 1996; Singh & Singh, 1998). *Capparis deciduas* and *Zingiber purpurem* have shown high oviposition inhibitory against *Bruchus chinensis* and Bruchids respectively (Bandara et al., 2005; Upadhyay, 2012). Ganapaty et al. (2004) reported that the extract of *Diospyros sylvatica* had repellent and toxic effects against *Odontotermes obesus*. Extracts of *Polygonum hydropiper* (L) and *Pogostemon paviflorus* (Benth) have shown high toxicity in *Odontotermes assamensis* (Holm) (Rahman et al., 2005). Concentrations determine the efficacy of botanical extracts. The oviposition rates of some Lepidopteran insects were not affected by low dose of *Azadirachtin* (Naumann & Isman, 1995; Saxena & Rembold, 1984). The oviposition punctures of *B. tryoni* were not influenced when apple fruits were treated with low dose of neem oil (Hidayat, Heather & Hassan, 2013). Extracts of *A. indica* have been found very effective against the *B*. *zonata, B. dorsalis*, and *B. olae* (Chen et al., 1996; Rehman et al., 2009a). However, to the best authors’ knowledge, no reports were given regarding the repellency of botanicals against *B. correcta*.

*Seriphidium brevifolium* Wall. ex DC. Ling & Y. R. Ling is a succulent plant and commonly used for the treatment of colds, flu and cough (Khan & Qaiser, 2006; Koop & Quinlivan, 2000). Water-soluble extracts of *Artemisia coerulescens* L. and *S. brevifolium* have been shown to have high toxicity against the larvae of *Culex pipiens* (Aly & Berger, 1996). However, *S. brevifolium* has not been tested against the *Bactrocera* species. The *Piper nigrum* L. is one of the most important aromatic spices and medicinal properties (Shanmugapriya et al., 2012). In recent years, the *P. nigrum* has been used as a repellent against different pests of Lepidoptera, Coleoptera and Diptera (Freeborn & Wymore, 1929; Lathrop & Keirstead, 1946; Trongtokit et al., 2005). However, no records were found against *Bactrocera* species. *Azadirachta indica* A. Juss (neem, nimtree or Indian lilac) has been tested against different *Bactrocera* species (Chen et al., 1996) but not against *B. correcta*. *Azadirachtin* is one of the most important active compounds that has been used as repellent and toxicant against a number of pests (Ilyas, Khan & Qadir, 2017; Isman, 2006). The *A. indica* could affect the life table traits and immunity of pests (Bezzar-Bendjazia et al., 2017; Isman, 2006; Schmutterer, 1990), especially Dipteran (Ilyas, Khan & Qadir, 2017). Seeds extracts of *A. indica* have been used against *B. cucurbitae* and *B. dorsalis* (Singh & Singh, 1998), but no records were published on the *B. correcta*. Quercetin is a phenolic component found in a number of plants (Anjaneyulu & Chopra, 2003). Quercetin is toxic and repellent to a number of Lepidopteran and Dipteran pests (Ahmad & Pardini, 1990; Hidayatulfathi et al., 2017; Selin-Rani et al., 2016). However, the repellency of quercetin against *B. dorsalis* and *B. correcta* species are still unknown.

However, to the best authors knowledge, *S. brevifolium*, *P. nigrum* and quercetin still have not been tested against *B. correcta*. However, seeds extract of *A. indica* have been tested against *B. dorsalis* (Hidayatulfathi et al., 2017; Selin-Rani et al., 2016), while not against the *B. correcta. S. brevifolium, P. nigrum* and *A. indica* are commonly cultivated plants in Asia especially in China. In addition, quercetin is a toxic phenolic compound and still not used against *B. dorsalis* and *B. correcta*. This study explains the repellency of seed extract of three botanicals (*S. brevifolium*, *P. nigrum* and *A. indica*) and a phenolic compound (quercetin) against the *B. dorsalis* and *B. correcta* on mangoes under laboratory conditions. This study will be useful for future use of these botanicals to *Bactrocera* species.

## Materials and Methods

### Insects

The population of both species (*B. dorsalis* and *B. correcta*) was reared in the controlled room (25 ± 2 °C, 60 ± 5% relative humidity, and a photoperiod of L:D 14:10 h) at the South China Agricultural University, Guangzhou, China. The temperature was controlled by the air-conditioner (Gree Electric Appliances, Inc. of Zhuhai, Zhuhai, China) and the humidity was maintained by a humidifier (Jaleel et al., 2018a, 2018b). Adult flies were reared in cages (30 × 30 × 30 cm) by providing water-soaked cotton wool in a box (12 × 6.8 × 7 cm), powdered yeast and sugar (2:1) in a petri-dish (6 × 1.5 cm). Larvae were reared on a semi-artificial diet described by Jaleel, Lu & He (2018).

### Mangoes

Mango (*M. indica* L. Hanana Datai Nong Mang, Yellow) fruits were purchased from a local market in Guangzhou, Guangdong Province, China. Fruits were bagged before the stage of ripening (to avoid the attack of wild fruit flies) in fields. To check the field infestation by wild fruit flies, six mangoes were randomly chosen and kept separately in a plastic jar (23.5 × 15.8 × 10 cm) containing a 3-cm layer of soil, either wild flies’ pupae were recovered or not. We did not recover pupae from these fruits. To find out the ripeness or sugar level, the following parameters were measured, total soluble salts (TSS) and the pericarp toughness or firmness of fruits were measured by handheld pocket refractometer pal-1 (ATAGO, PR-101a, Brix 0–45%; Tokyo Tech., Tokyo, Japan) and TMS-Pro texture analyzer (FTC-TV, Rainsville, AL, USA) with probe (one mm diameter) respectively. The hole diameter by the female fly of *B. dorsalis* has been reported 0.1–0.2 mm on mango fruit. Measurements were taken and recorded at three different locations on mango fruit. Fifteen replications were done (Balagawi et al., 2005; Díaz-Fleischer & Aluja, 2003; Jaleel et al., 2018a; Rattanapun, Amornsak & Clarke, 2009).

### Plants

Seeds of *S. brevifolium* were collected at Halqa 2 Skardu (35.18°N, 75.37°E), Skardu Baltistan, Pakistan. Seeds of *A. indica* were collected at Multan, Punjab, Pakistan (30.16°N, 71.52°E). Seeds of *P. nigrum* were purchased from the local market in Guangzhou, China. The fine powder of quercetin (95%) was bought from Sigma–Aldrich Co. (St. Louis, MI, USA).

### Preparation of plant extracts solutions

Five hundred grams of seeds of *S. brevifolium*, *P. nigrum* and *A. indica* were dried in the electric oven (DHG-9240A; Shanghai Qi Xin Ke Xue Yiqi, Co., Ltd., Shanghai, China) at 50 °C for 24 h and ground into fine powder in an electronic blender (OPY-908; Zhongshan Opaye Industry Co., Ltd., Zhongshan, China). Two hundred grams were measured and soaked in a conical flask having acetone (97%) with a ratio of 1:2 (w/v). All the filtrates were combined together and allowed to evaporate at the rotary evaporator. Each mixture was stirred by the ultrasound method for 30 min and then placed in the dark for 24 h. Then, the supernatant was filtered with a double layer of Whatman filter paper no. 42. The mentioned procedure was repeated thrice to gain maximumly extractable. To make homogenous and concentrated paste, the filtered solutions were evaporated using a rotary evaporator (RE-52AA; Shandong, Biotechnology Co., Ltd., Taian City, China) under reduced pressure and temperature at 55 °C, then made solvent free in a vacuum desiccator. Then, all extracts were preserved at 4 °C until used (Ilyas, Khan & Qadir, 2017).

Formulation of *S. brevifolium*, *P. nigrum*, *A. indica* and quercetin were prepared using the acetone (20%) and by mixing five drops of tween—80 (0.001%) as emulsifier with five ml of plant extract, then acetone mixture was added up to 100 ml to obtain 5% concentration of each plant extract. The other desired dilutions (2.5% and 1%) were prepared from each stock solution (5%).

### Data recording

**Figure 1:**
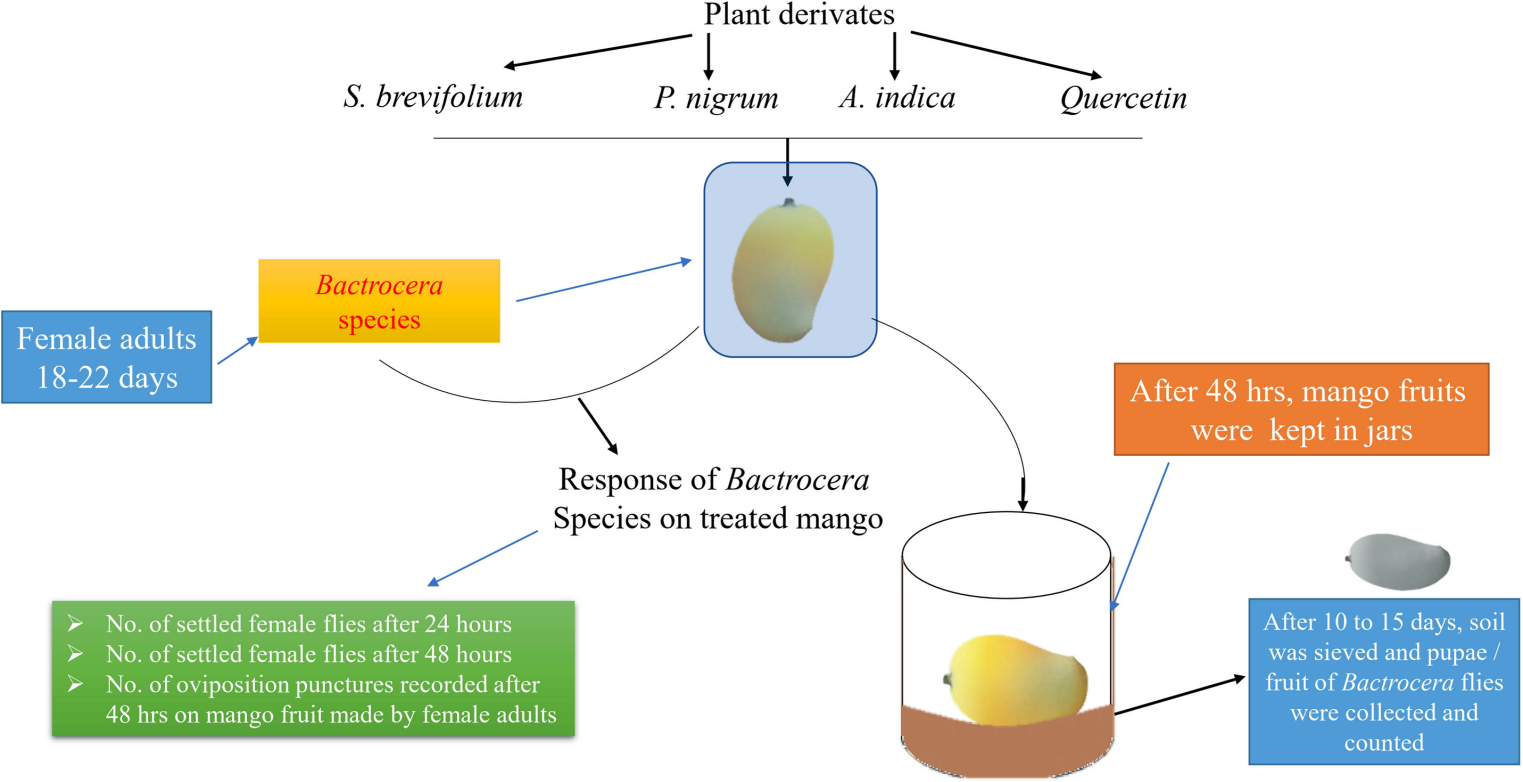
Experimental layout.

Treated and untreated mangoes were placed into the cages (45 × 40 × 40 cm) in a free-choice test. In this study, the treated mangoes with one concentration and untreated mango were put in a separate cage. Fifteen mangoes were used for each concentration. Each mango was considered a replication. The experimental design was arranged according to a Completely Randomized Design. The experimental layout has shown in Fig. 1. Twenty gravid female flies of *B. dorsalis* or *B. correcta* (18–22 days of age) were released into each cage (treatment). After 24 h, the number of female flies on the surface of treated and untreated mangoes was recorded. Observations were conducted for 10 h; each fruit was observed for 5 min in an hour. Then, after 48 h, the number of female flies on the fruit surface was also recorded using the same method. Then treated and untreated mangoes were removed from the cages, and the number of oviposition punctures/fruit was counted. Then both treated and untreated mangoes were separately kept in plastic jars (12 × 6.0 × 12 cm) having 3 cm soil layer on the bottom for pupation and covered with a muslin cloth. After 10–15 days, the soil was sieved, and pupae/mango of both species were counted.

### Statistical analysis

Number of visits, ovipositional punctures, and pupae of both flies to plants were summarized as the percentage of visiting flies (the number of flies visiting treated mangoes divided by the total number of visiting flies on both treated and untreated mangoes), the oviposition punctures on the treated mangoes (the number of oviposition punctures on the treated mangoes divided by the total number of oviposition punctures on both treated and untreated mangoes), and the percentage of pupae developed in the treated mangoes (the number of pupae developed in the treated mangoes divided by the total number of pupae in both treated and untreated mangoes). Data were normally distributed; the percentage data were arcsine square root transformed prior to analysis, if necessary. One-way analysis of variance was used to analyze the number of visits (%), ovipositional punctures (%) and pupae (%). All data analyses were carried out using SPSS version 22.0 (International Business Machines Corp., Armonk, NY, USA).

## Results

### Fruit characteristics

**Figure 2:**
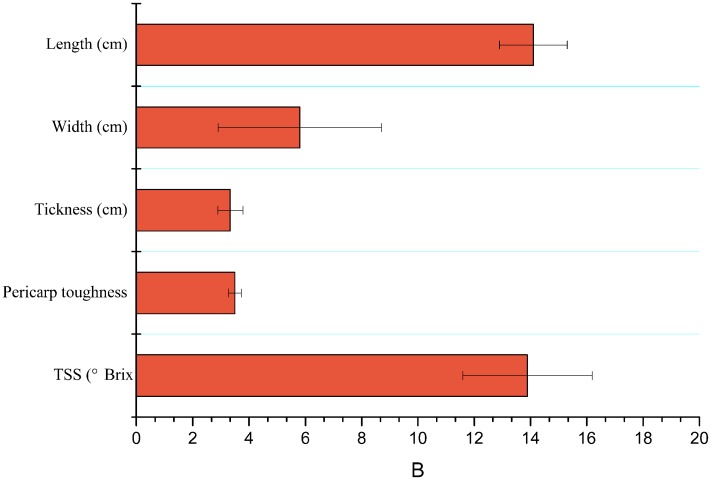
Mean (±SE) of physicochemical properties of mango fruits (*n* = 15). TSS, total soluble salts.

Characteristics like length (cm), width (cm), thickness (cm), TSS or brix firmness/hardness (N) of mango fruits are shown in Fig. 2.

### Visits after 24 h

**Figure 3:**
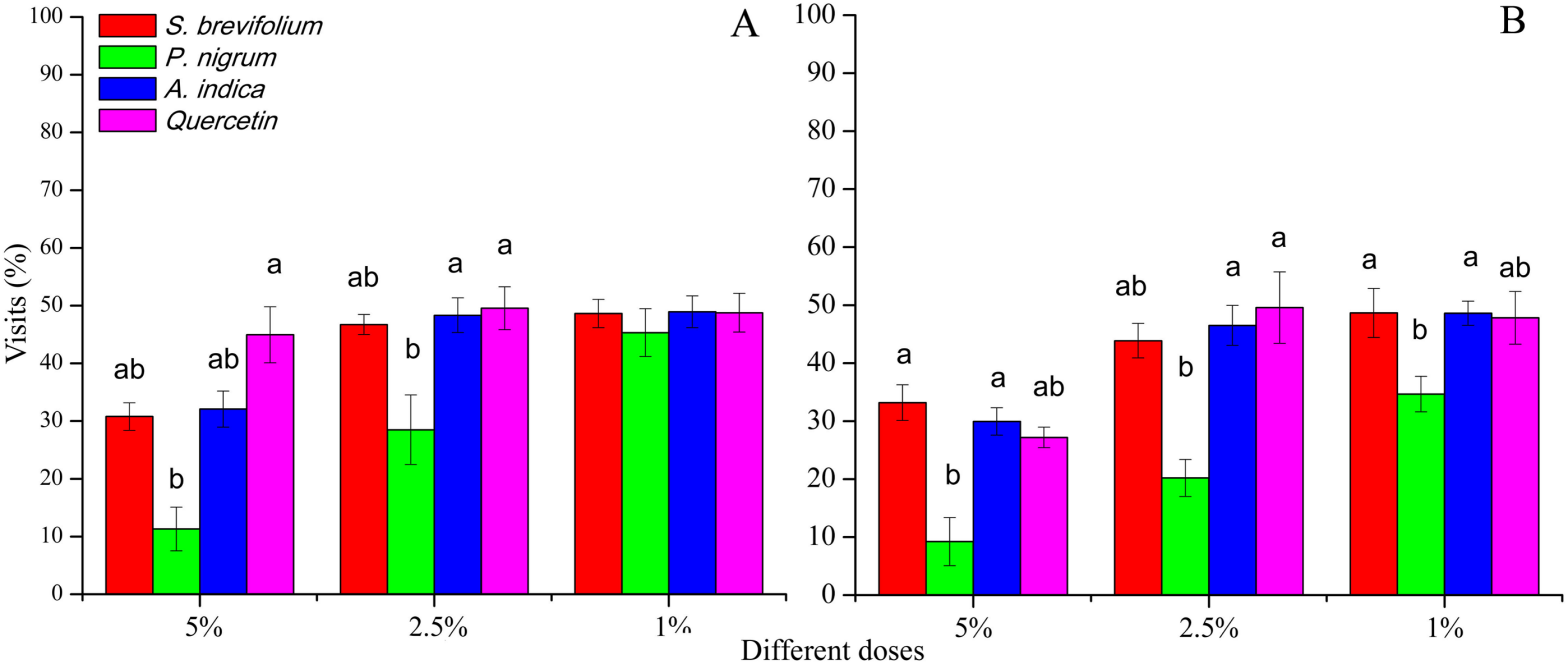
Visit (%) of female adults of *B. dorsalis* (A) and *B. correcta* (B) on treated mango fruits with the concentrations of 5%, 2.5% and 1% of four botanicals after 24 h. Within a botanical concentrations, the means with different letters are significantly different (Kruskal–Wallis ****one-way ANOVA, at *P* < 0.05, all-pairwise comparisons test of homogenous group).

Female *B. dorsalis* visits (%) after 24 h were minimum on mangoes treated by the *P. nigrum* at 5% (*F*_3,__20_ = 21.30, *P* < 0.001) and 2.5% (*F*_3, 20_ = 11.60, *P* < 0.001) concentrations as compared to the other botanicals (*S. brevifolium*, *A. indica* and quercetin) and their concentrations (5% and 2.5%) (Fig. 3A). In case of *B. correcta*, visits (%) after 24 h were less on mangoes treated by the *P. nigrum* at all concentration for example, 5% (*F*_3, 20_ = 16.20, *P* < 0.001), 2.5% (*F*_3, 20_ = 9.92, *P* < 0.001), and 1% (*F*_3, 20_ = 6.74, *P* = 0.003) as compared to rest of three botanicals (quercetin, *S. brevifolium* and *A. indica*) and their concentrations (5%, 2.5% and 1%) (Fig. 3B).

### Visits after 48 h

**Figure 4:**
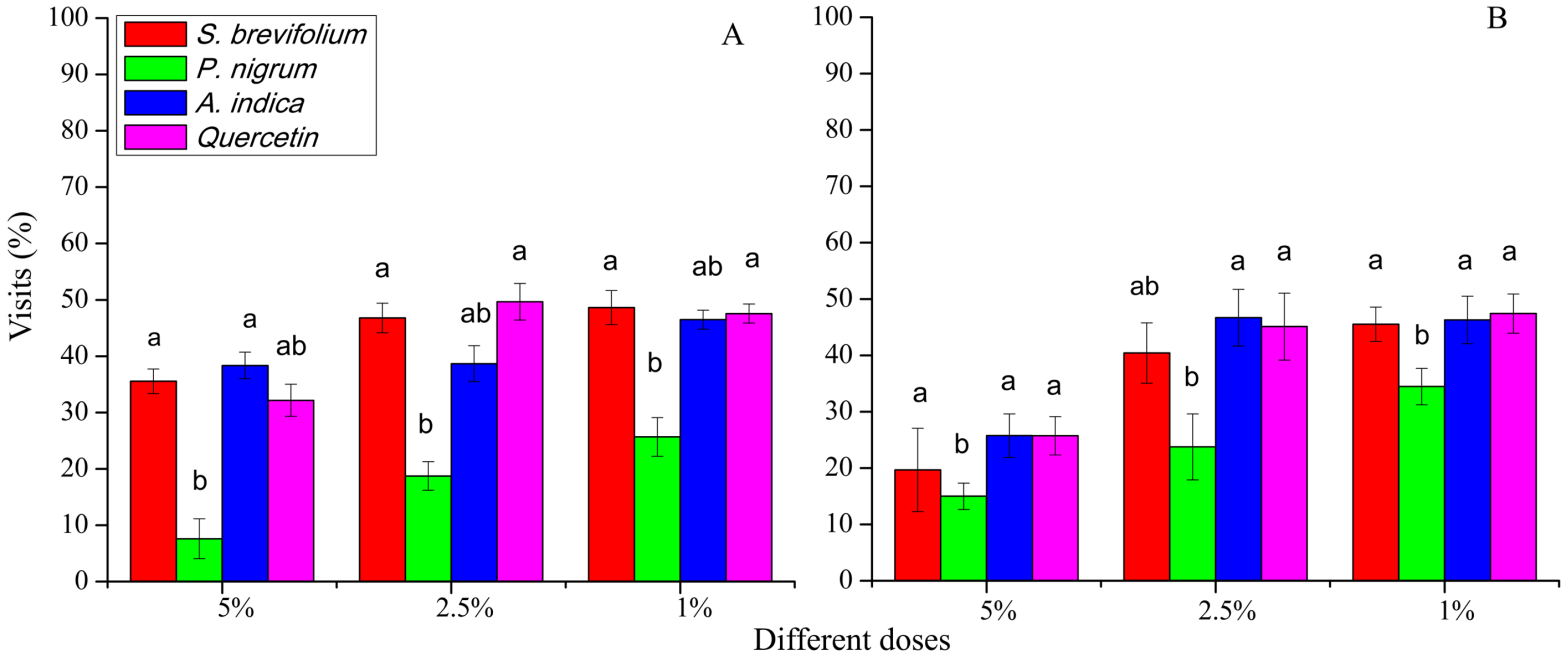
Visit (%) of female adults of *B. dorsalis* (A) and *B. correcta* (B) on treated mango fruits with the concentrations of 5%, 2.5% and 1% of four botanicals after 48 h. Within botanical concentrations, the means with different letters are significantly different (Kruskal–Wallis ****one-way ANOVA, at *P* < 0.05, all-pairwise comparisons test of homogenous group).

Visits (%) after 48 h done by female *B. dorsalis* adults were less on the *P. nigrum* mangoes treated at all concentration (5%; *F*_3, 20_ = 13.40, *P* < 0.001, 2.5%; *F*_3, 20_ = 27.20, *P* < 0.001 and 1%; *F*_3, 20_ = 10.40, *P* < 0.001) as compared to other three botanicals (*S. brevifolium*, *A. indica* and quercetin) and their concentrations (5%, 2.5% and 1%) (Fig. 4A). In case of *B. correcta*, visits (%) after 48 h were minimum on the mangoes treated by the *P. nigrum* at 5% (*F*_3, 20_ = 2.90, *P* = 0.045), and 2.5% concentrations (*F*_3, 20_ = 6.49, *P* = 0.003) concentrations as compared to rest of three botanicals (*S. brevifolium*, *A. indica* and quercetin) and their concentrations (5% and 2.5%) (Fig. 4B).

### Oviposition punctures

**Figure 5:**
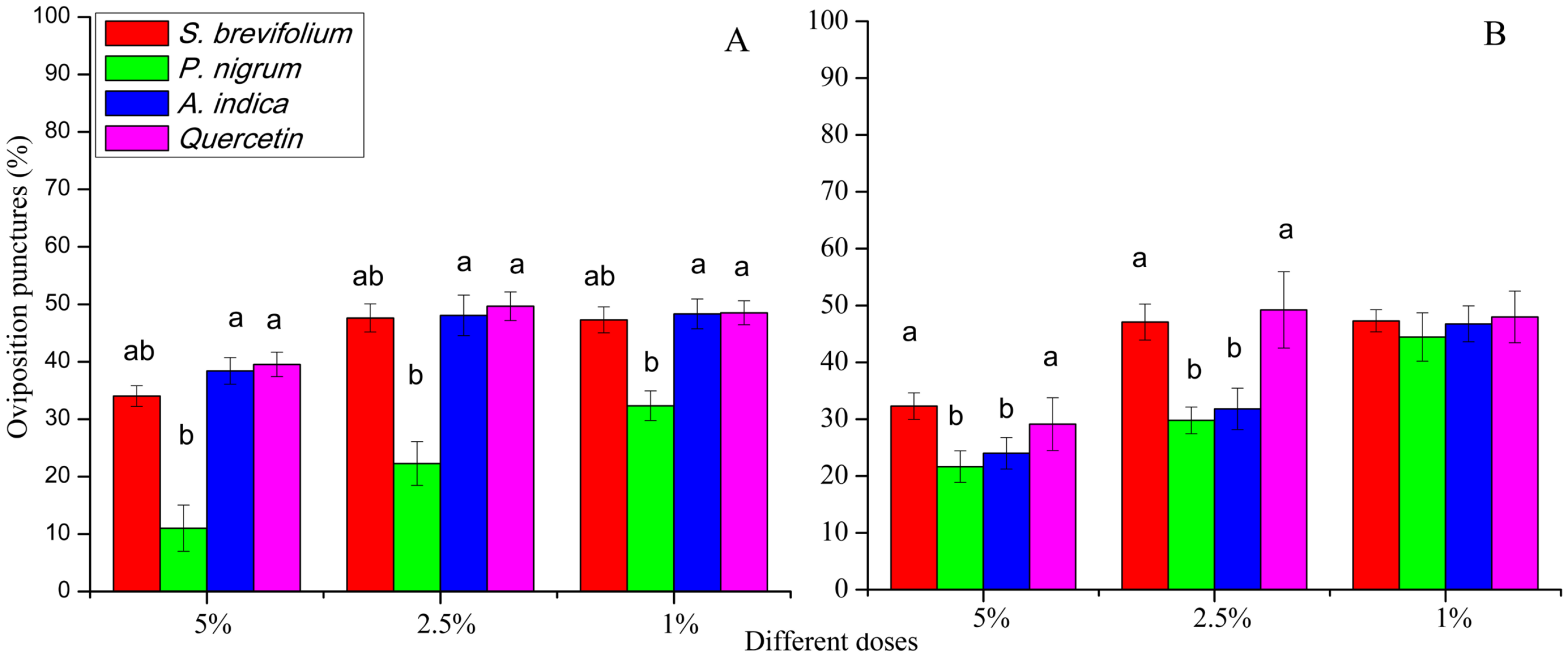
Oviposition punctures (%) by female flies of *B. dorsalis* (A) and *B. correcta* (B) on treated mango fruits with four botanicals and their concentrations. Within a botanical concentrations, the means with different letters are significantly different (Kruskal–Wallis ****one-way ANOVA, at *P* < 0.05, all-pairwise comparisons test of homogenous group).

The oviposition punctures made by female flies of *B. dorsalis* were significantly reduced on mangoes treated by *P. nigrum* at all concentrations for example, 5% (*F*_3, 20_ = 16.30, *P* < 0.001), 2.5% (*F*_3, 20_ = 11.60, *P* < 0.001), and 1% (*F*_3, 20_ = 4.46, *P* = 0.015) as compared to other three botanicals (*S. brevifolium*, *A. indica* and quercetin) and their concentrations (5%, 2.5% and 1%) respectively (Fig. 5A). The oviposition punctures (%) done by female flies of *B. correcta* were minimum on the mangoes treated by *P. nigrum* at 5% (*F*_3, 20_ = 3.76, *P* = 0.027) and 2.5% (*F*_3, 20_ = 3.03, *P* = 0.034) concentrations as compared to other three botanicals (*S. brevifolium*, *A. indica* and quercetin) and their concentrations (5% and 2.5%) (Fig. 5B).

### Pupae

**Figure 6:**
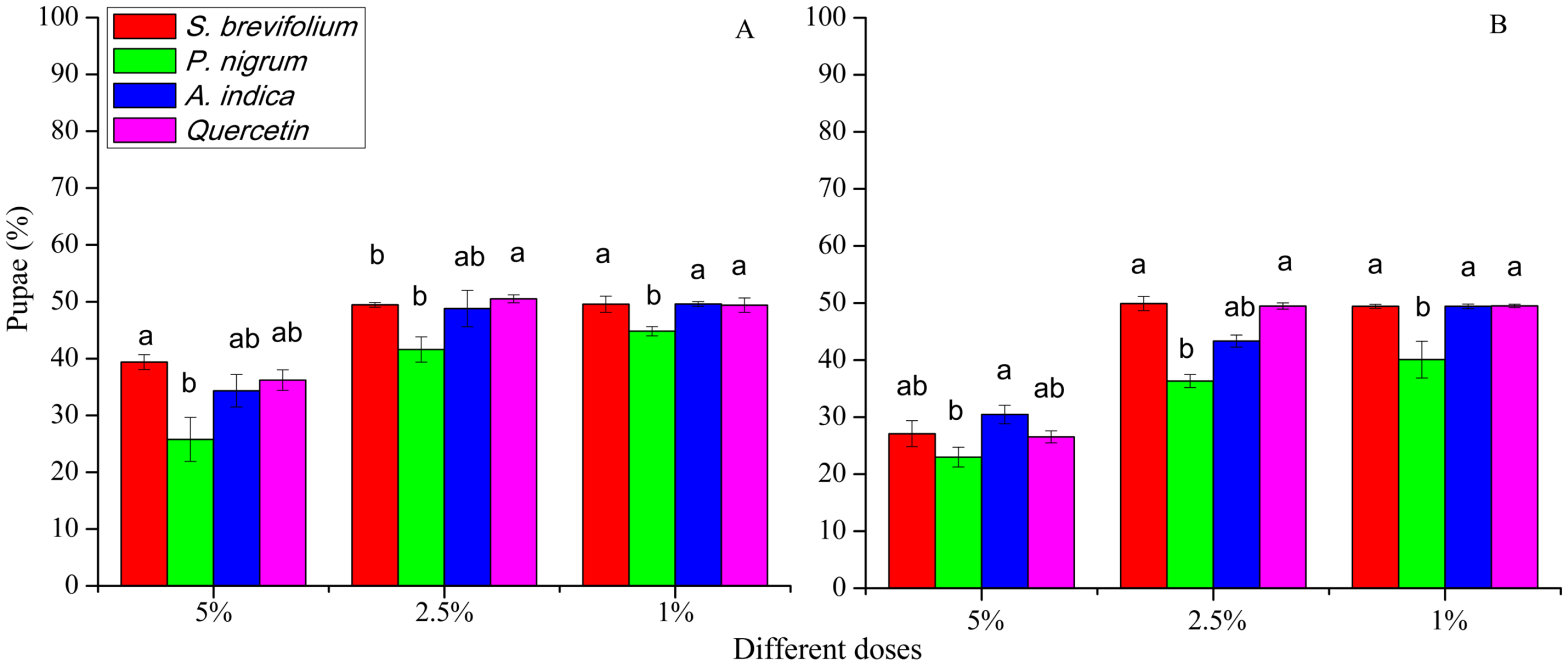
Pupae (%) of *B. dorsalis* (A) and *B. correcta* (B) from treated mango fruits with four botanicals and their concentration. Within a botanical concentrations, the means with different letters are significantly different (Kruskal-Wallis ****one-way ANOVA, at *P* < 0.05, all-pairwise comparisons test of homogenous group).

The retrieved pupae (%) of *B. dorsalis* were minimum from mangoes treated by the *P. nigrum* at all concentrations for example, 5% (*F*_3, 20_ = 33.30, *P* < 0.001), 2.5% (*F*_3, 20_ = 15.20, *P* < 0.001), and 1% (*F*_3, 20_ = 9.25, *P* < 0.001) as compared to other three botanicals (*S. brevifolium*, *A. indica* and quercetin) and their concentrations (5%, 2.5% and 1 %) (Fig. 5A). Similarly, in the case of *B. correcta*, the pupae (%) were less from mangoes treated by the *P. nigrum* mangoes at all concentrations for example, 5% (*F*_3, 20_ = 6.09, *P* = 0.004), 2.5% (*F*_3, 20_ = 39.5, *P* < 0.001), 1% (*F*_3, 20_ = 8.83, *P* < 0.001) as compared to other three botanicals (*S. brevifolium*, *A. indica* and quercetin) and their concentrations (5%, 2.5% and 1%) (Fig. 6B). However, extracts of *S. brevifolium*, *P. nigrum*, *A. indica* and quercetin were effective to reduce the visits, ovipositional punctures, and pupae of both species. Among all plants, the *P. nigrum* was the more active repellent to *B. correcta* and then *B. dorsalis*.

## Discussion

This study first time describes settling and ovipositional response *B. dorsalis* and *B. correcta* on the mangoes treated by *S. brevifolium, P. nigrum, A. indica* and quercetin. The *P. nigrum* was the best repellent to *B. correcta* and then *B. dorsalis*.

The repellency of botanicals extract usually depends on the methods of extraction and solvent type. Siddiqi et al. (2006) concluded that the acetone extract was highly repellent to *B. zonata*. Rehman et al. (2009a) have been used six botanicals extract (*Acorus calamus* L., *Citrullus colocynthis* L., *Curcuma longa* L., *Saussurea lappa, Valeriana jatamansi* Jones and *Peganum harmala* L.) against the *B. zonata* and concluded that extract of *C. longa* and *P. harmala* were highly repellent against the peach fruit fly. In this study, *P. nigrum* was the best deterrent against both species than those of the other three botanicals.

The *P. nigrum* was found to be the best repellent against *Sitophilus zeamais* Motsch (Ishii, Matsuzawa & Vairappan, 2010). *Peganum harmala* at 2% concentration had higher repellency rate against the *B. oleae* (Rehman et al., 2009b). In this study, the visits and oviposition puncture made by females of *B. dorsalis* and *B. correcta* were significantly less on mangoes treated by the *P. nigrum* as compared to other three botanicals. The *A. indica* have been found to be very effective to reduce the oviposition rate of *B*. *zonata, B. dorsalis* and *B. olae* (Chen et al., 1996; Rehman et al., 2009a). The oviposition rates of some Lepidopteran insects were not affected on *Azadirachtin* treated plants at the low doses (Naumann & Isman, 1995; Saxena & Rembold, 1984). The oviposition punctures have not been influenced in female *B. tryoni* adults when apple fruits were treated with neem oil (10 mL/L) in both choice and no-choice experiments (Hidayat, Heather & Hassan, 2013). In this study, there was no significant difference in oviposition punctures (done by female flies of *B. dorsalis* and *B. correcta*) between treated and untreated mangoes at low concentration (1%) of *S. brevifolium*, *A. indica* and quercetin except *P. nigrum*. Our study supports the findings of Saxena & Rembold (1984), Naumann & Isman (1995) and Hidayat, Heather & Hassan (2013). *Azadirachtin* has been found to be most effective in reducing the oviposition rates of oriental fruit flies on the melons treated at high doses (Khan, Hossain & Islam, 2007). The *E. camaldulensis* had best repellent effect against *B. zonata* and significantly reduced the pupal development of *B. zonata* (Rehman et al., 2009a). In our study, the number of pupae of both species were lower in the mangoes treated by *P. nigrum* as compared to the other three botanicals.

## Conclusion

This study only explained the repellency of four botanicals after 24 and 48 h, However, the repellency of botanicals as natural pesticides remained for a longer period. This study contains solid data to support future works on the repellency of *S. brevifolium*, *P. nigrum*, *A. indica* and quercetin. In conclusion, *P. nigrum* was the best repellent in comparison to *S. brevifolium*, *A. indica* and quercetin against *B. dorsalis* and *B. correcta*. More work is needed to find out active repellent and deterrent components/compounds in the *S. brevifolium*, *P. nigrum* and *A. indica* through GCMS and LC-MS. Their modes of action may require further explorations against both *Bactrocera* species as well as their efficacy at farm and orchard.

## Supplemental Information

10.7717/peerj.8537/supp-1 Supplemental Information 1 Highlights.

10.7717/peerj.8537/supp-2 Supplemental Information 2 Raw data.

## Additional Information and Declarations

### Data Availability

The following information was supplied regarding data availability: The raw data are available in a Supplemental File.
