# Supplementary material for: Evaluating the repellent effect of four botanicals against two Bactrocera species on mangoes
Source: PeerJ. 2020 Mar 4;8:e8537. doi: 10.7717/peerj.8537 (PMC7060752; doi:10.7717/peerj.8537)
Supplement: Supplemental Information 1 [file peerj-08-8537-s001.docx]

**Highlights**

- Study focus the settling and ovipositional behavior of *Bactrocera dorsalis and B. correcta*
- Study focus the repellency of four botanicals (*Seriphidium brevifolium, Piper nigrum, Azadirachta indica,* and quercetin) against *Bactrocera dorsalis and B. correcta* that have not been tested before.
- Extracts of *S. brevifolium, P. nigrum, A. indica* and quercetin were effective to reduce the visits, ovipositional punctures, and pupae of both species.
- *Piper nigrum* was more effective against both species*.*
